# Age-Related Biology of Early-Stage Operable Breast Cancer and Its Impact on Clinical Outcome

**DOI:** 10.3390/cancers13061417

**Published:** 2021-03-19

**Authors:** Binafsha M. Syed, Andrew R. Green, Emad A. Rakha, David A.L. Morgan, Ian O. Ellis, Kwok-Leung Cheung

**Affiliations:** 1 School of Medicine, University of Nottingham, Derby DE22 3DT, UK; binafsha.syed@lumhs.edu.pk (B.M.S.); andrew.green@nottingham.ac.uk (A.R.G.); emad.rakha@nottingham.ac.uk (E.A.R.); ian.ellis@nottingham.ac.uk (I.O.E.); 2 Medical Research Centre, Liaquat University of Medical & Health Sciences, Jamshoro 71000, Pakistan; 3 Department of Oncology, Nottingham University Hospitals, Nottingham NG5 1PB, UK; dalmorgan@me.com

**Keywords:** biology, breast cancer, older women

## Abstract

**Simple Summary:**

Breast cancer incidence not only increases with advancing age but also changes its biology. This study was conducted to understand aging related change in the biological characteristics of breast cancer. The results highlighted that the change occurs in a gradual fashion, where 40 years and 70 years become the milestones for significant difference. Breast cancer in patients <40 years showed aggressive characteristics while at 70 years and above they are more indolent. The molecular pattern between 40 years and 70 years appears to be a transition from aggressive to less aggressive phenotypes. This change in the biology of the breast cancer significantly influences clinical outcome.

**Abstract:**

As age advances, breast cancer (BC) tends to change its biological characteristics. This study aimed to explore the natural progression of such changes. The study included 2383 women with clinically T0-2N0-1M0 BC, managed by primary surgery and optimal adjuvant therapy in a dedicated BC facility. Tissue micro-arrays were constructed from their surgical specimens and indirect immunohistochemistry was used for analysis of a large panel (*n* = 16) of relevant biomarkers. There were significant changes in the pattern of expression of biomarkers related to luminal (oestrogen receptor (ER), progesterone receptors (PgR), human epidermal growth factor receptor (HER-2), E-cadherin, MUC1, bcl2 CK7/8, CK18 and bcl2) and basal (CK5/6, CK14, p53 and Ki67) phenotypes, lymph node stage, histological grade and pathological size when decade-wise comparison was made (*p* < 0.05). The ages of 40 years and 70 years appeared to be the milestones marking a change of the pattern. There were significantly higher metastasis free and breast cancer specific survival rates among older women with ER positive tumours while there was no significant difference in the ER negative group according to age. Biological characteristics of BC show a pattern of change with advancing age, where 40 years and 70 years appear as important milestones. The pattern suggests <40 years as the phase with aggressive phenotypes, >70 years as the less aggressive phase and 40–70 years being the transitional phase.

## 1. Introduction

Breast cancer is the most common malignancy among females, and the leading cause of death worldwide [1]. With advancing age, not only does the incidence of breast cancer rise, but also there appears to be a change in the biological characteristics. Starting from the basic morphological features, older women tend to have a relatively higher rate of lobular carcinoma, and a lower rate of more aggressive types like medullary carcinoma [2,3,4]. Older women tend to have more grade I and II tumours as compared to the younger population where they are more likely to have grade III tumours [3,5,6]. According to the existing evidence, ER expression rises and the expression of HER2 and Ki67 declines with advancing age [2,7]. Therefore, it is generally accepted that older women tend to have less aggressive tumours when compared to their younger counterparts [2,5]. It is debatable that aging directly causes these differences, nevertheless a whole spectrum of biological alterations are likely to occur, which in turn results in a change in the clinical behaviour of the cancer [8]. However, there is limited information available to understand the natural progression of the pattern of biological characteristics with age. One method which could potentially tease out the pattern is the analysis of expression of biomarkers across different age extremes. To date however this has only been attempted in very few studies [9,10], and they tend to include a small number of markers (i.e., mainly including conventional markers such as oestrogen receptor (ER), progesterone receptors (PgR), human epidermal growth factor receptor (HER-2), Ki67 and bcl2, which might not be sufficient to delineate any patterns. Furthermore, the data were mostly collected retrospectively from multiple centres using different laboratory methodologies further limiting their potential significance. 

This study therefore took a stepwise approach and aimed to:

1. Explore the natural progression, if any, of the change in the biological characteristics according to age as a whole. Given that the ER positive and ER negative phenotypes show completely distinct clinical behaviours, their biological characteristics were also compared and assessed according to age.
2. Compare clinical outcomes of the patients in different age groups, if significant changes in their biological characteristics were found.
3. Compare the treatment patterns, if significant differences in clinical outcome were found.

## 2. Results

There were 2383 patients including: younger Nottingham Tenovus series—(*n* = 1808, age range 18–70 years), and Elderly Primary Breast cancer series—(*n* = 575, age range 70–91 years). They were divided in decade wise fashion as follows: ≤30 (*n* = 19), 31–40 (*n* = 144), 41–50 (*n* = 496), 51–60 (*n* = 599), 61–70 (*n* = 551), 71–80 (*n* = 484) and ≥81 (*n* = 90). 

### 2.1. Biological Features

#### 2.1.1. Overall Pattern of Biomarkers

There were significant “step changes” according to age in the pattern of positivity of ER (*p* < 0.001), PgR (*p* = 0.003), HER4 (*p* < 0.001), E-cadherin (*p* < 0.001), Ki67 (*p* < 0.001), p53 (*p* = 0.003), CK5/6 (*p* < 0.001), CK7/8 (*p* = 0.006), CK14 (*p* < 0.001), CK17 (*p* < 0.03), CK18 (*p* < 0.001) and bcl2 (*p* < 0.001). The ages of 40 years and 70 years were shown to be the significant milestones when these “step changes” occurred (Table 1). Lymph node stage (*p* = 0.01), pathological size of tumour (*p* < 0.001) and histological grade (*p* < 0.001) also showed a significant pattern of change according to age (Table 1).

**Figure 1:**
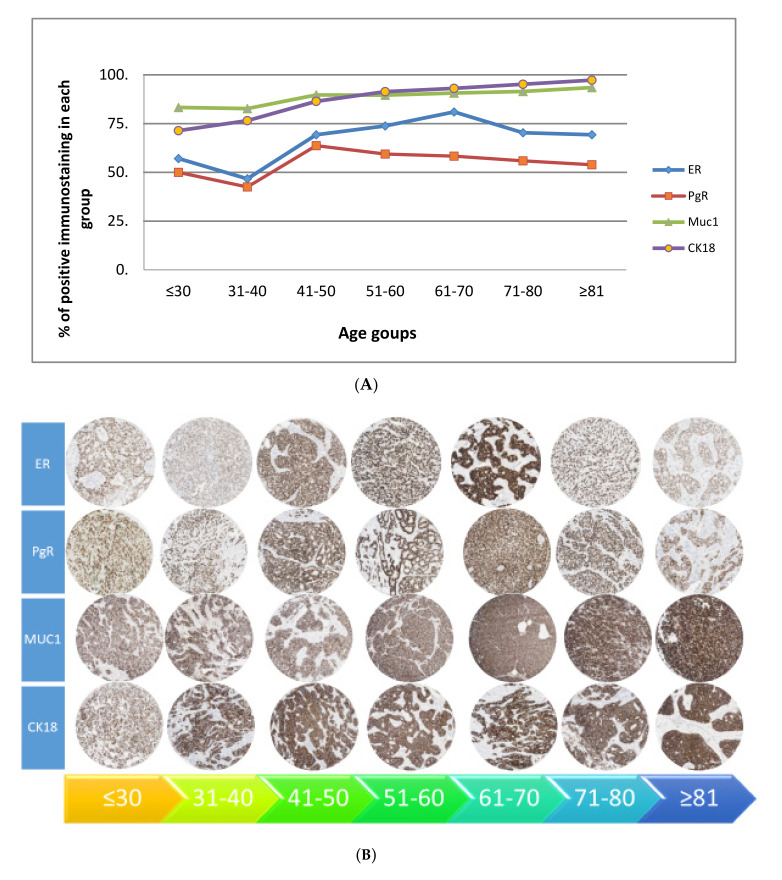

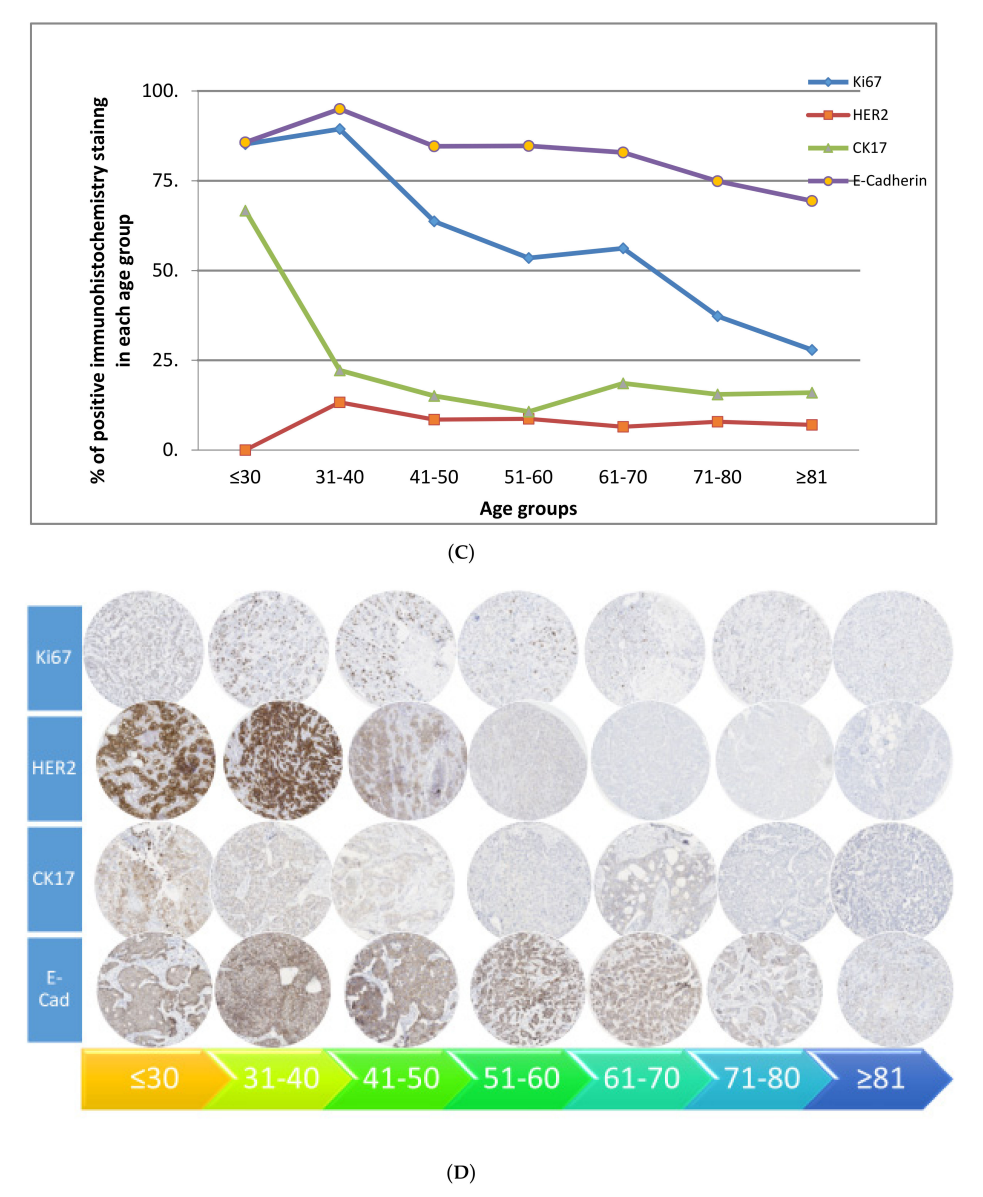

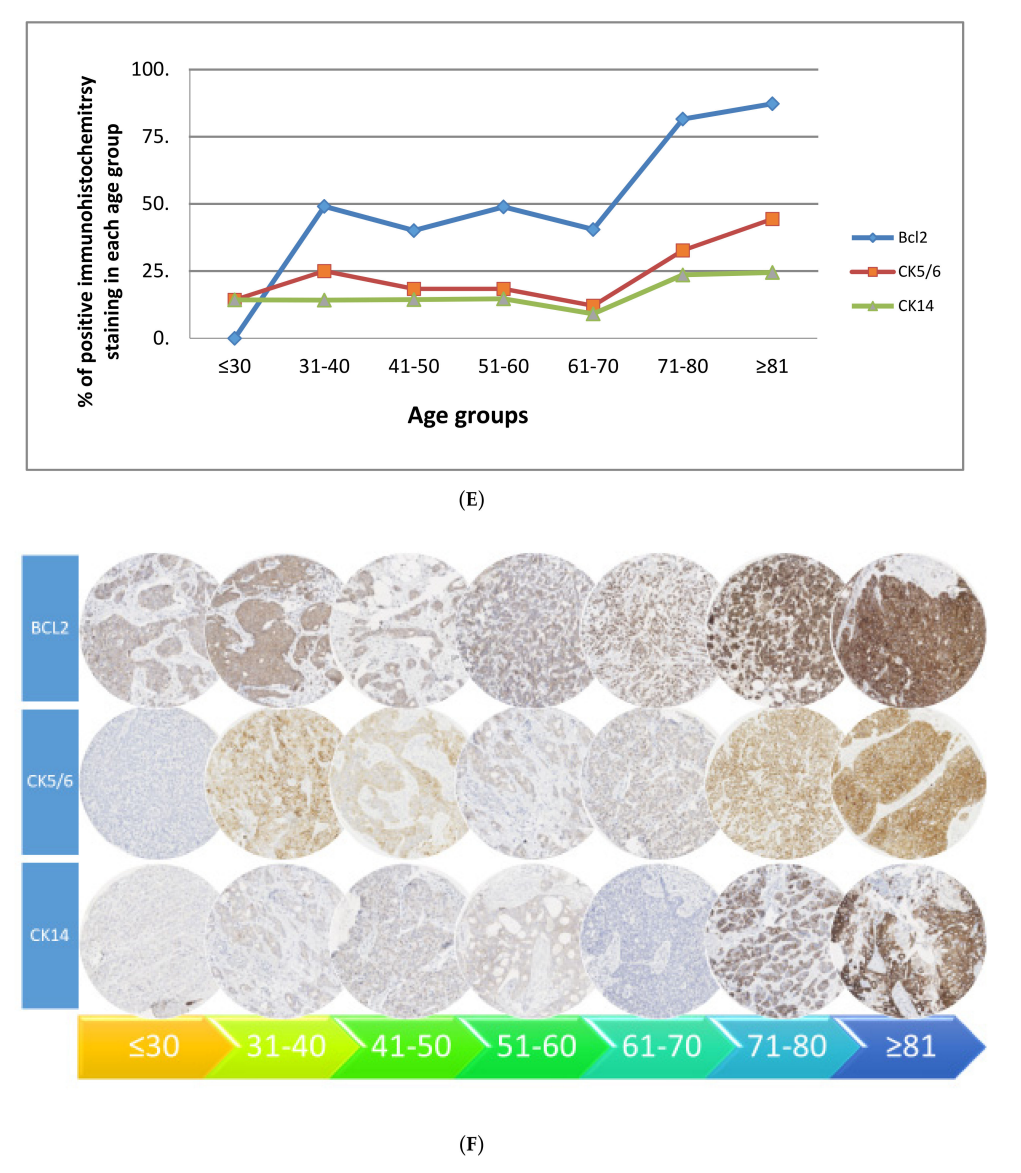

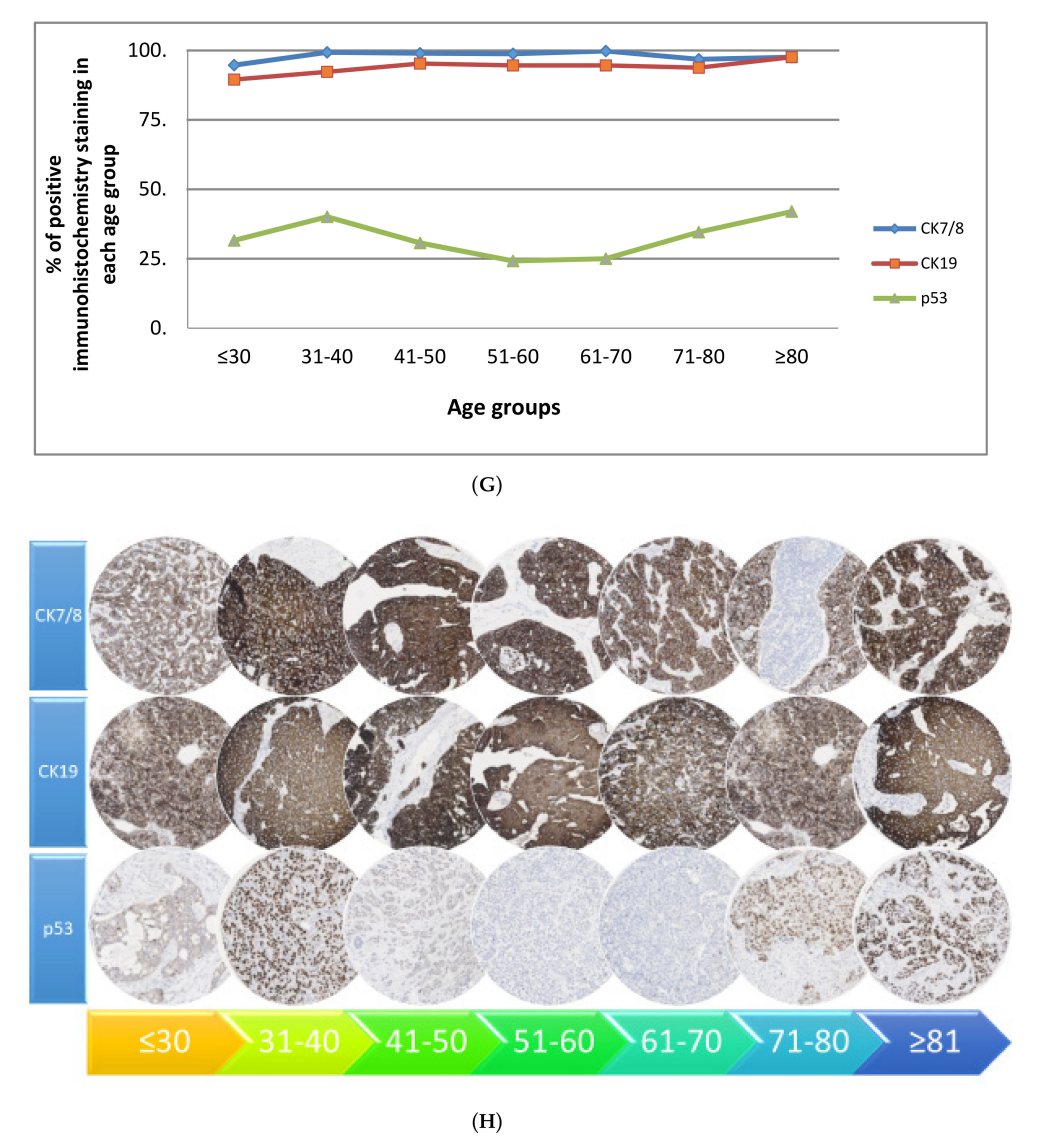
(**A**) Pattern of the expression of biomarkers with age stratification in early operable primary breast cancer treated by surgery—significant rise at 40 years. (**B**) Pattern of the expression of biomarkers with age stratification in early operable primary breast cancer treated by surgery—significant rise at 40 years (at nanozoomer magnification ×20). (**C**) Pattern of the expression of biomarkers with age stratification in early operable primary breast cancer treated by surgery—significant decline at 40 years. (**D**) Pattern of the expression of biomarkers with age stratification in early operable primary breast cancer treated by surgery—significant decline at 40 years (at nanozoomer magnification ×20). (**E**) Pattern of the expression of biomarkers with age stratification in early operable primary breast cancer treated by surgery—significant rise at 70 years. (**F**) Pattern of the expression of biomarkers with age stratification in early operable primary breast cancer treated by surgery—significant rise at 70 years (at nanozoomer magnification ×20). (**G**) Pattern of the expression of biomarkers with age stratification in early operable primary breast cancer treated by surgery—two peaks. (**H**) Pattern of the expression of biomarkers with age stratification in early operable primary breast cancer treated by surgery—two peaks (at nanozoomer magnification ×20).

The overall age stratified expression of biomarkers showed four patterns (Figure 1):

4. A gradual rise starting at 40 years—ER, PgR, MUC1 and CK18.
5. A gradual decline starting at 40 years—Ki67, HER2, CK17 and E-cadherin.
6. A rise at 70 years—bcl2, CK5/6 and CK14.
7. Two peaks—CK7/8, CK19 and p53 (i.e., peak at 40 years then decline, rising again at 70 years).

Based on these patterns, the patients could be categorized into three distinct age groups i.e., <40, 41–69 and ≥70 years. Immunohistochemical expression is presented in Figure 1 for key patterns. 

#### 2.1.2. Pattern of Biomarkers According to ER Status

##### ER Positive Group

**Figure 2:**
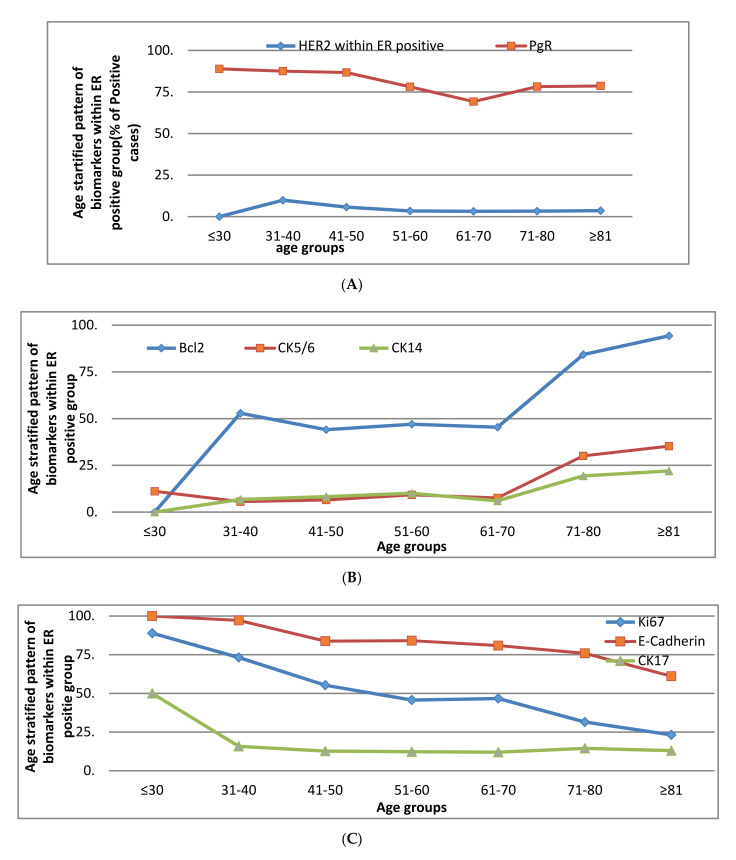

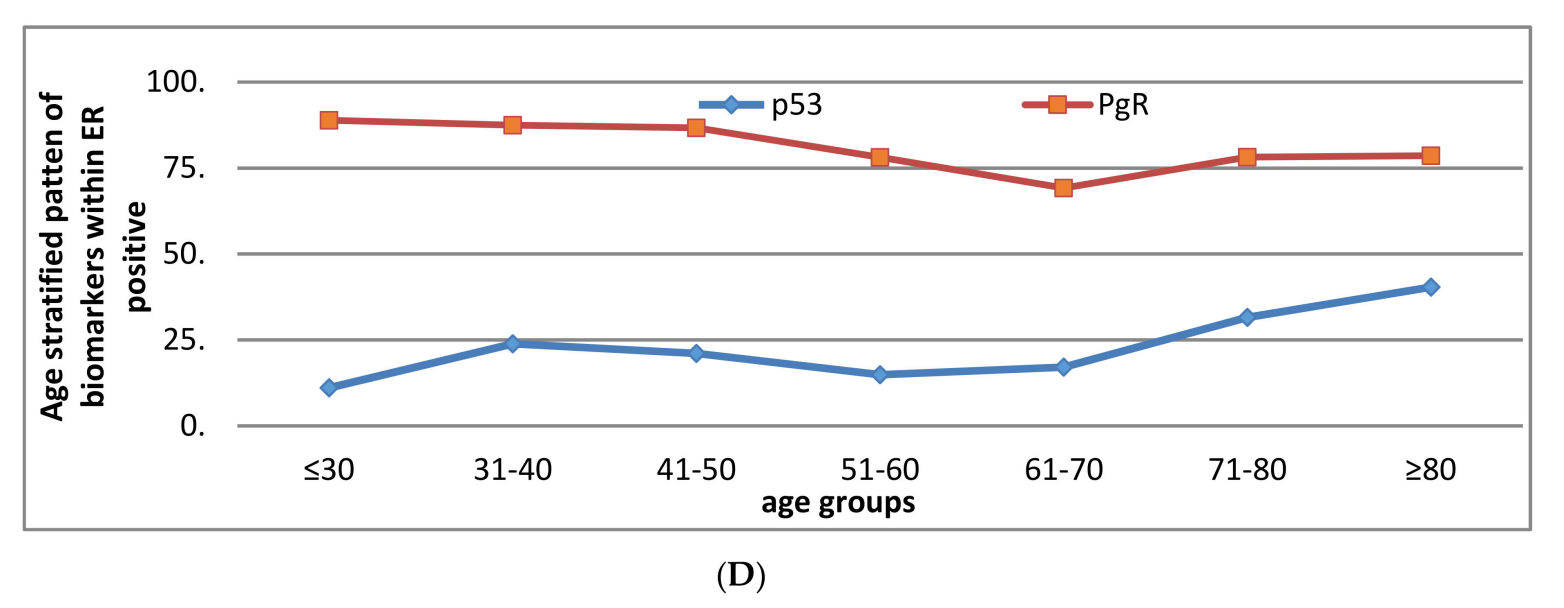
(**A**) Pattern of the expression of biomarkers with age stratification in ER positive early operable primary breast cancer treated by surgery—decline at 40 years. (**B**) Pattern of the expression of biomarkers with age stratification in ER positive early operable primary breast cancer treated by surgery—rise at 70 years. (**C**) Pattern of the expression of biomarkers with age stratification in ER positive early operable primary breast cancer treated by surgery—consistent decline. (**D**) Pattern of the expression of biomarkers with age stratification in ER positive early operable primary breast cancer treated by surgery—two peaks.

The following pattern was observed within the ER positive group (Figure 2):

a. A rise at 40 years—HER2
b. A rise at 70 years—bcl2, CK5/6, CK14
c. Decline with advancing age—Ki67, E-cadherin
d. Two peaks—p53 and PgR (at 40 and 70 years)

However, MUC1, CK18, CK7/8 and CK19 remained unchanged throughout (more than 80% of tumours were positive).

e. ER negative group

**Figure 3:**
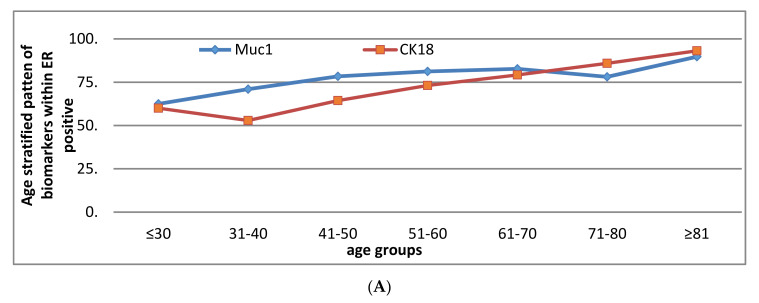

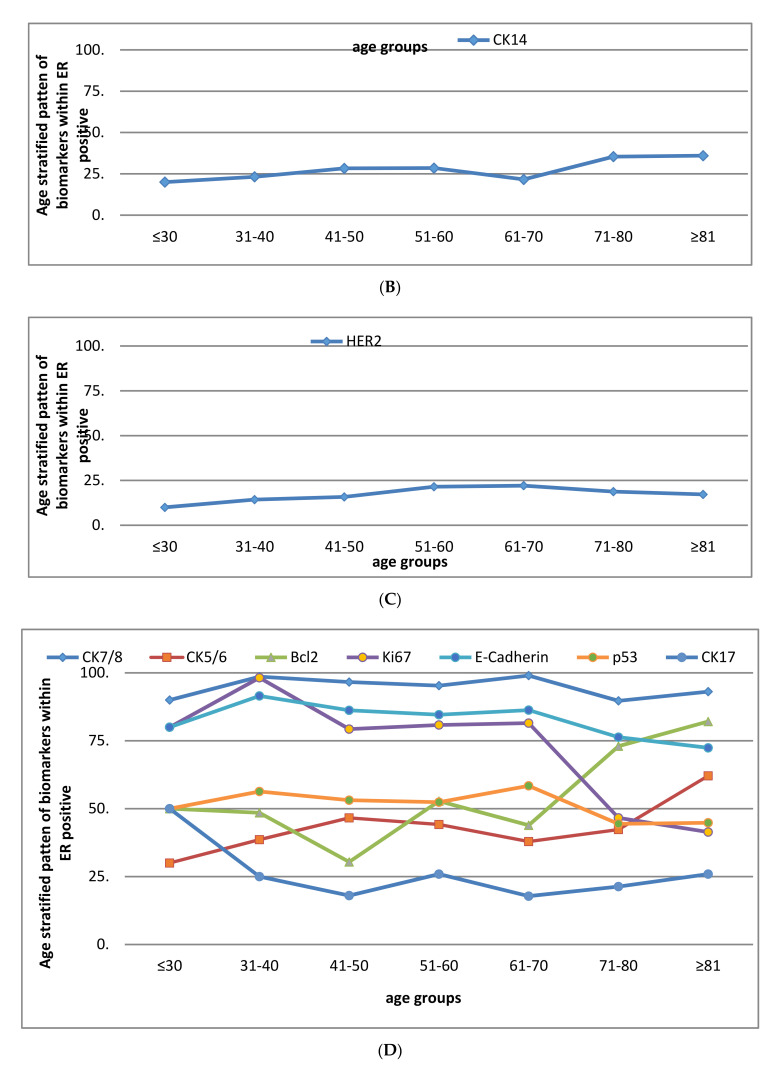
(**A**) Pattern of the expression of biomarkers with age stratification in ER negative early operable primary breast cancer treated by surgery—gradual rise. (**B**) Pattern of the expression of biomarkers with age stratification in ER negative early operable primary breast cancer treated by surgery—rise at 70 years. (**C**) Pattern of the expression of biomarkers with age stratification in ER negative early operable primary breast cancer treated by surgery—decline at 70. (**D**) Pattern of the expression of biomarkers with age stratification in ER negative early operable primary breast cancer treated by surgery— two peaks.

The following pattern was observed within the ER negative group (Figure 3).

a. A rise at 40 years—MUC1 and CK18
b. A rise at 70 years—CK14
c. Rise at 30 years then decline at 70 years—HER2
d. Two peaks—Bcl2, CK5/6, Ki67, CK7/8, E-cadherin, p53 and CK17 (at 40 and 70 years).

However, PgR remained unchanged within the ER negative group (i.e., 93.9% negative).

### 2.2. Clinical Outcome

#### Breast Cancer Specific and Metastasis Free Survival Rates

Older women showed significantly higher 5-year Breast Cancer Specific Survival (BCSS) (≤40 years = 75%, 41–69 years = 86%, ≥70 years = 90%, *p*-value 0.04, Figure S1). While comparing patients with ER negative and ER positive tumours in the same age groups, those with ER positive tumours showed significantly higher BCSS compared to those with ER negative tumours in the 41–70 and >70 years age groups. However, when just those with ER positive tumours were investigated in different age groups, older women had significantly higher BCSS, as compared to their younger counterparts (Figure S1). 

Metastasis free survival was also significantly higher among older women with ER positive tumours (*p* = 0.03) as compared to younger age groups. Among patients with ER negative tumours across all age groups, BCSS and metastasis free survival both remained non-significant (Table 2). 

### 2.3. Treatment Pattern

Most of the younger patients received more “aggressive” treatment including adjuvant chemotherapy and radiotherapy while a considerable number of older women received surgery without adjuvant chemotherapy. Adjuvant endocrine therapy was used considerably more frequently among older women with ER positive tumours, as compared to younger patients (<40 years = 32%, 41–70 years = 41%, ≥70 years = 64%) A summary of the treatment pattern is given in Table 3. 

## 3. Discussion

The results of the study suggest that the biology of breast cancer alters in a gradual fashion in relation to the age at the time of diagnosis. From the age stratified pattern, three biological groups were identified, ≤40, 41–70 and >70 years of age. The pattern persisted in both the ER positive and negative subgroups.

Tissue aging carries a high risk of cancer development due to a number of factors; for instance, the long-term endogenous oestrogen exposure, reduction in the DNA repairing ability of cells (high rate of p53 mutation), and a reduction in immunity with advancing age may contribute to the process of carcinogenesis [10,11,12,13]. However, it is not yet fully understood how advancing age produces such change in the biological characteristics of the tumours. 

In our study, the biological characteristics of breast cancer appeared to show a pattern of alteration with increasing age, as a whole group and also when they were subdivided into ER positive and negative subgroups. The ER expression itself showed a rise with advancing age, which is in keeping with the available literature [2,9,10,14]. However, its slight decline at 70 years in this series could be due to natural selection of relatively more ER negative tumours for surgery, as the study was based on biomarker measurements only in patients undergoing surgery, thereby excluding those receiving primary endocrine therapy (most likely ER positive cases). 

The pattern of biomarkers showing a consistent rise, a consistent fall, an initial rise then a fall or vice versa is in keeping with the available literature [9] and also the age stratified pattern of ER, PgR, HER2, luminal and basal cytokeratins, Ki67 and EGFR is consistent with the existing literature [2,9,10,15]. However, the pattern of markers with age stratification according to their ER status has never been reported before. In addition, our study includes a large panel of biomarkers with long term clinical outcomes making our study novel. The pattern of p53 appeared in contrast to the results of other studies [2,9,10,15] where there was a consistent reduction in positive expression; however, the results of our study support the concept of reduction in the DNA repairing ability of the cells with advancing age. In our study we looked at the protein expression of p53 which if present suggests the presence of the mutant p53 gene. Within our data the pattern of p53 was different in ER positive and ER negative groups, with decreasing numbers of positive p53 cases in the ER negative subgroup and increasing numbers in the ER positive subgroup, where there is again a possibility that the ER positive tumours might have pathological relation to somatic p53 mutations in older women as a result of long standing oestrogen stimulation for cell division. Nevertheless, in future, in vitro or cell line studies will be required to analyse the role of p53 in breast cancer development and progression in older women.

Overall the pattern of biological markers showed three different groups based on their biological characteristics: (i) “≤40 years” where there was low ER, PgR, luminal cytokeratins, bcl2 and high Ki67, HER2 and p53 expression; (ii) exactly the opposite pattern was seen in older women “>70 years”, and (iii) the third group “between 40 and 70 years”, suggesting a “transition” stage. The age-related biological pattern is in keeping with the general impression of aggressive tumour biology in very young patients and less aggressive biology in older women. Another interesting point worth noting about the pattern of age groups is that patients <40 years are expected to be pre-menopausal. The older age group (>70 years) are invariably post-menopausal while those between 40 and 70 should include pre-, peri- and early post-menopausal women. This pattern supports the hypothesis that circulating oestrogens may play a key role in determining the biology of breast cancer. In addition, when closely looking at the carcinogenesis which involves proto-oncogenes (which promote cell division) and tumour suppressor genes (which suppress cell division), the younger age group theoretically appears to be linked with proto-oncogenes while the older age with faulty suppressor mechanism. However, little is known about the precise relationship between carcinogenesis and age. 

The clinical outcome data reported in our study suggests that older women achieved better survival as compared to the younger population where there was poor metastasis-free and breast cancer specific survival. This is in line with the aggressive tumour biology in younger patients (Table 1). There is an interesting finding of significantly better clinical outcome among older women again suggesting a favourable biology. There was no significant difference in the survival of patients with ER negative tumours based on age though younger patients received more aggressive therapy (i.e., adjuvant chemotherapy was given to the majority of patients of <40 years while none of the older (>70 years) patients received it (Table 3). Among patients with ER positive tumours, the use of chemotherapy was reduced with advancing age as only two patients in the older age group received such treatment, while endocrine therapy was widely used: over 64% of older women (>70) received such treatment. The treatment pattern is in line with the existing literature which suggests a less aggressive treatment pattern in older women. Thus, the difference in the clinical outcome with better survival in older women appears to be influenced by less aggressive biology. 

Our study is strengthened by incorporating a large sample size with long term follow-up including all age groups. Such a large dataset comparing biological characteristics across all age groups from a single centre following the same methods is reported for the first time. The tumour samples were prospectively collected and tissue micro-arrays were constructed with biomarker measurements using a centralised and standardised protocol from a single laboratory. However, the study has the limitation of not including needle core biopsy specimens thus a considerable number of the older women who received non-surgical therapy were excluded giving a potential bias towards having proportionally more ER negative tumours in the older age group. 

## 4. Materials and Methods 

### 4.1. Patients

This study included women with early-stage operable breast cancer (clinically T0–2N0-1M0) undergoing primary surgery without any prior intervention, from two series of well characterised patients managed at a dedicated facility following the same management protocol at any given time, with good quality surgical resection samples available for tissue micro-array (TMA) construction. The women ≤70 years of age were from the Nottingham primary breast cancer series (*n* = 1808) [16] and women ≥70 years from the Nottingham elderly primary breast cancer series (*n* = 575) [17]. For this study patients were grouped in decades according to their age at diagnosis, i.e., ≤30, 31–40, 41–50, 51–60, 61–70, 71–80 and ≥80 years. The study was approved by the Research Ethics Committee at the Nottingham City Hospital under ethical approval number C2020313. The tumour samples were collected historically before the Human Tissue Act came in. The Research Ethics Committee considered that patient consents were deemed not necessary.

### 4.2. Tumour Analysis 

Tissue microarrays were constructed from formalin-fixed paraffin-embedded surgical specimens [18]. Briefly, Haematoxylin and Eosin was used to identify the most representative part of the tumour, and 0.6 mm-diameter cores of the representative part of the tumour blocks were implanted in the TMA blocks using Beecher’s manual tissue microarrayer (MP06 Beecher Instruments Inc., Sun Prarie, WI, USA). One most representative core was taken for each TMA block. Biological analysis of the tumours was carried out using indirect immunohistochemistry (IHC) by StreptAvidin Biotin Complex and EnVision methods as described [19]. Immunohistochemical scoring for tissue staining was done by using McCarty’s immunohistochemical scoring (H-score) (range 0–300) [20] and the percentage of positive cells was counted. The H-scoring system considers both number of cells showing staining and intensity of the staining in each cell. X-tile Bio-informatics software was used to define cut-offs to define positive expression of the biomarkers [21]. Cut-off values of the protein expression was previously defined [7] and Table S1 is attached.

Immunohistochemical analysis of the TMAs was carried out for a panel of 16 biomarkers, including hormone receptors (ER and PgR), HER2, Ki67, luminal (CK7/8, CK18,CK19, MUC1, E-cadherin and bcl2 and HER4) and basal (BRCA1, CK5/6, CK14, CK17 and p53) markers.

Given the distinct differences in the clinical behaviour between ER positive and ER negative tumours, both groups were compared in terms of their biological pattern and clinical outcome.

### 4.3. Clinical Outcome

The clinical outcome of the patients was analysed in terms of breast cancer specific survival and metastases free survival, defined as the time duration from date of diagnosis till death and confirmation of distant metastases, respectively.

### 4.4. Statistical analysis

The data was analysed using Statistical Package for Social Sciences (SPSS version 18.0, Chicago, IL, USA). The Kruskal–Wallis test was applied to evaluate the groups to see if there were any dominant patterns. This was followed by the application of Mann–Whitney U test to locate the difference, which was further confirmed by Chi-square test. Survival analysis was done by using Kaplan–Meier methods with application of Log-rank and generalised Wilcoxon tests as appropriate. A *p*-value <0.05 was considered significant.

## 5. Conclusions

In conclusion, we confirm that the biology of breast cancer alters with age, with a gradual rise of good prognostic markers illustrating a characteristic pattern of three groups with cut-off age points at 40 years and 70 years. The clinical outcome was better among older patients in general, those with ER positive tumours having significantly better survival as compared to their younger counterparts within same age group while there was no significant difference in the clinical outcome in ER negative subgroups. The treatment pattern showed more aggressive adjuvant therapy given in younger patients when compared to the older patients. The study thus suggests a less aggressive tumour biology with advancing age resulting in better survival. Further studies are required to understand cellular mechanisms and age induced changes in the intracellular environment leading to the development and/or progression of breast cancer in order to optimise the management of women with different tumour phenotypes.

## Figures and Tables

**Table 1:** Age standardised pattern of biological markers in early operable primary breast cancer.

Biomarker (n)	≤30 n (%)	31–40 n (%)	41–50 n (%)	51–60 n (%)	61–70 n (%)	71–80 n (%)	≥81 n (%)	*p*-Value
Lymph node stage								
1	7(50)	62 (50.8)	281 (62.0)	378 (64.1)	395 (68.6)	192 (57.1)	38 (57.6)	0.01
2	4 (28.6)	44 (36.1)	133 (29.4)	168 (28.5)	133 (23.1)	106 (31.5)	21 (31.8)	
3	3 (21.4)	16 (13.1)	39 (8.6)	44 (7.5)	48 (8.3)	38 (11.3)	7 (10.6)	
Histological Grade								
1	0	5 (4.1)	83 (18.3)	131 (22.2)	110 (19.0)	51 (12.1)	13 (12.9)	<0.001
2	2 (14.3)	25 (20.5)	140 (30.9)	200 (34.0)	209 (36.1)	166 (39.2)	46 (45.5)	
3	12 (85.7)	92 (75.4)	230 (50.8)	258 (43.8)	260 (44.9)	206 (48.7)	42 (41.6)	
Pathological size								
0.1–3.0 cm	9 (64.3)	100 (82.0)	402 (88.9)	533 (90.5)	537 (92.7)	377 (80.2)	87 (77.0)	<0.001
3.1–5.0 cm	3 (21.4)	16 (13.1)	41 (9.1)	45 (7.6)	40 (6.9)	84 (17.9)	19 (16.8)	
>5.0 cm	2 (14.3)	6 (4.9)	9 (2.0)	11 (1.9)	2 (0.3)	9 (1.9)	7 (6.2)	
ER positive (*n* = 2333)	8 (57.1)	57 (46.7)	314 (69.3)	436 (73.8)	470 (81.0)	322 (70.3)	79 (69.3)	<0.001
PgR (*n* = 2278)	7 (50.0)	51 (42.5)	281 (63.7)	339 (59.4)	327 (58.3)	255 (55.9)	62 (53.9)	0.003
HER2 (*n* = 2297)	0	16 (13.3)	37 (8.5)	50 (8.7)	37 (6.5)	37 (7.9)	8 (7.0)	0.232
Ki67 (*n* = 2020)	12 (85.7)	84 (89.4)	239 (63.7)	255 (53.5)	251 (56.2)	183 (37.3)	34 (27.9)	<0.001
P53 (*n* = 2267)	3 (21.4)	53 (44.2)	133 (30.2)	152 (26.6)	127 (22.6)	156 (34.8)	43 (39.1)	<0.001
Bcl2 (*n* = 1221)	0	28 (49.1)	73 (40.1)	110 (48.9)	90 (40.5)	345 (81.6)	96 (87.3)	<0.001
Muc1 (*n* = 1962)	10 (83.3)	86 (82.7)	330 (89.7)	413 (89.6)	419 (90.7)	409 (91.5)	101 (93.5)	0.133
BRCA1 (*n* = 1979)	11 (84.6)	83 (80.6)	313 (82.8)	393 (83.4)	403 (86.1)	152 (34.5)	36 (34.3)	<0.001
CK5/6 (*n* = 2274)	2 (14.3)	30 (25.0)	82 (18.4)	105 (18.4)	69 (12.1)	146 (32.7)	48 (44.4)	<0.001
CK7/8 (*n* = 2296)	13 (92.6)	119 (99.2)	441 (98.9)	573 (99.0)	571 (99.7)	440 (97.1)	108 (97.3)	0.006
CK14 (*n* = 2193)	2 (14.3)	17 (14.2)	64 (14.4)	84 (14.7)	51 (9.1)	91 (23.6)	25 (24.5)	<0.001
CK17 (*n* = 1160)	2 (66.7)	8 (22.2)	24 (15.1)	32 (10.7)	80 (18.6)	17 (15.5)	186 (16.0)	0.035
CK18 (*n* = 2176)	10 (71.4)	91 (76.5)	361 (86.4)	490 (91.4)	501 (93.1)	417 (95.2)	110 (97.3)	<0.001
CK19 (*n* = 2292)	12 (85.7)	111 (92.5)	425 (95.3)	547 (94.3)	538 (94.6)	424 (94.0)	109 (97.3)	0.481
E-Cadherin (*n* = 2289)	12 (85.7)	115 (95.0)	374 (84.6)	488 (84.7)	469 (82.9)	344 (74.9)	77 (69.4)	<0.001

**Table 2:** Clinical outcome of early operable primary breast cancer—comparison among age groups based on oestrogen receptor status.

Outcome Measure	≤40 Years	41–70 Years	≥70 years	*p*-Value
5-year Metastases free survival (%)				
ER positive	68	85	87	0.003
ER negative	68	70	71	0.81
5-year Breast cancer specific survival (%)				
ER positive	85	89	94	0.03
ER negative	70	73	75	0.88

**Table 3:** Pattern of treatment of early operable primary breast cancer—comparison among age groups based on oestrogen receptor status.

	Surgery Only	Surgery Followed by Chemotherapy + /− Radiotherapy	Surgery Followed by Endocrine Therapy + /− Radiotherapy	Surgery Followed by Radiotherapy
ER negative age groups *n* (%)				
≤40	5 (6.7)	61 (81.3)	0	9 (12.0)
41–70	59 (16.3)	124 (34.3)	108 (29.8)	71 (19.6)
>70	122 (72.2)	0	18 (10.7)	29 (17.2)
ER positive age groups n (%)				
≤40	14 (1)	29 (36.7)	17 (21.5)	19 (24.1)
41–70	326 (28.5)	94 (8.2)	463 (40.5)	260 (22.7)
>70	116 (29.1)	2 (0.5)	257 (64.4)	24 (6.0)

## Data Availability

No new data were created or analyzed in this study. Data sharing is not applicable to this article.

## Supplementary Materials

The following are available online at https://www.mdpi.com/2072-6694/13/6/1417/s1, Figure S1: (a) Breast cancer specific survival—comparison of age groups <40, 41–70 years and >70 years, (b) Breast cancer specific survival Oestrogen receptor positive—comparison of age groups <40, 41–70 years and >70 years, (c) Breast cancer specific survival in Oestrogen receptor negative—comparison of age groups <40, 41–70 years and >70 years, (d) Breast cancer specific survival <40 years—comparison of oestrogen negative and oestrogen receptor positive groups, (e) Breast cancer specific survival 41–70 years—comparison of oestrogen negative and oestrogen receptor positive groups, (f) Breast cancer specific survival >70 years—comparison of oestrogen negative and oestrogen receptor positive groups, g. Local recurrence free survival—comparison of age groups <40, 41–70 years and >70 years, (h) Local recurrence free survival <40 years—comparison of oestrogen negative and oestrogen receptor positive groups, (i) Local recurrence free survival 41–70 years—comparison of oestrogen negative and oestrogen receptor positive groups, (j) Local recurrence free survival >70 years—comparison of oestrogen negative and oestrogen receptor positive groups, (k) Local recurrence free survival Oestrogen receptor positive—comparison of age groups <40, 41–70 years and >70 years, (l) Local recurrence free survival Oestrogen receptor negative—comparison of age groups <40, 41–70 years and >70 years, (m) Regional recurrence free survival Oestrogen receptor negative—comparison of age groups <40, 41–70 years and >70 years, (n) Regional recurrence free survival Oestrogen receptor positive—comparison of age groups <40, 41–70 years and >70 years, (o) Metastases free survival Oestrogen receptor negative—comparison of age groups <40, 41–70 years and >70 years, (p) Metastases free survival Oestrogen receptor positive—comparison of age groups <40, 41–70 years and >70 years; Table S1: Immunostaining cut-offs and cellular localization.
