# Supplementary material for: Age-Related Biology of Early-Stage Operable Breast Cancer and Its Impact on Clinical Outcome
Source: Cancers (Basel). 2021 Mar 19;13(6):1417. doi: 10.3390/cancers13061417 (PMC8003777; doi:10.3390/cancers13061417)
Supplement: Supplementary file 1 [file cancers-13-01417-s001.pdf]

Supplementary Materials:

Table S1: Immunostaining cut-offs and cellular localization

S #	Molecular marker	Cut-off (% of cells stained)	Cellular location
1	ER	1	Nuclear
2	PgR	1	Nuclear
3	HER2	3+	Cell membrane
4	Ki67	10	Nuclear
5	P53	5	Nuclear
6	Bcl2	1	Cytoplasm
7	CK7/8	1	Cytoplasm
8	CK18	1	Cytoplasm
9	CK19	1	Cytoplasm
10	MUC1	10	Cytoplasm
11	E-Cadherin	30	Cell membrane
12	HER4	1	Cell membrane
13	CK5/6	1	Cytoplasm
14	CK14	1	Cytoplasm
15	CK17	1	Cytoplasm
16	BRCA1	30	Nuclear

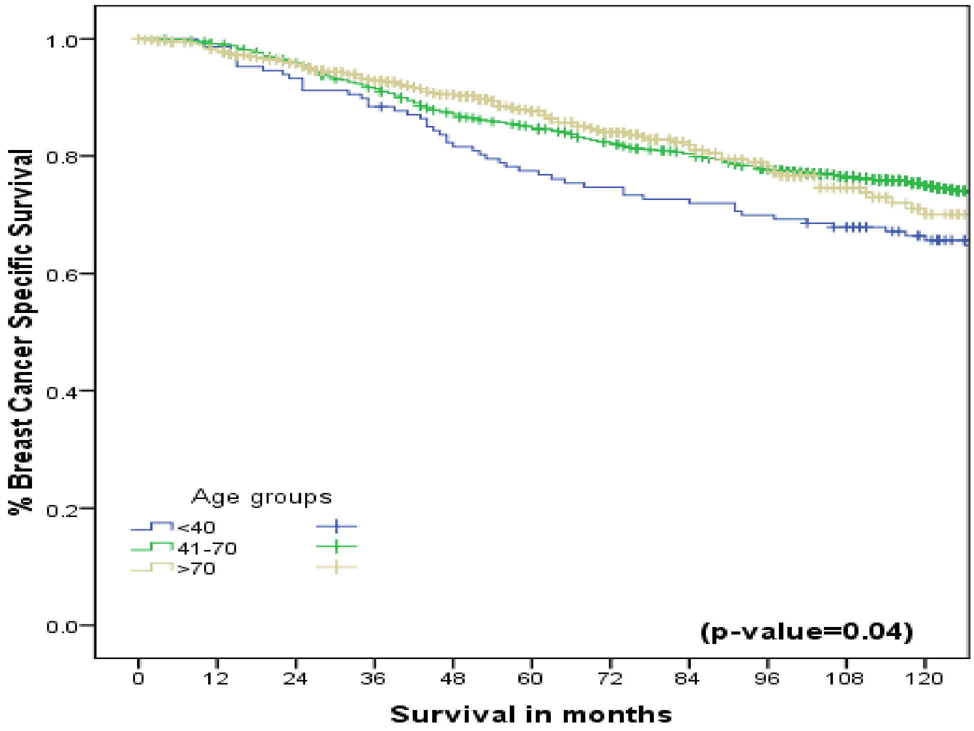

S1- a. Breast cancer specific survival – comparison of age groups <40, 41-70 years and >70 years

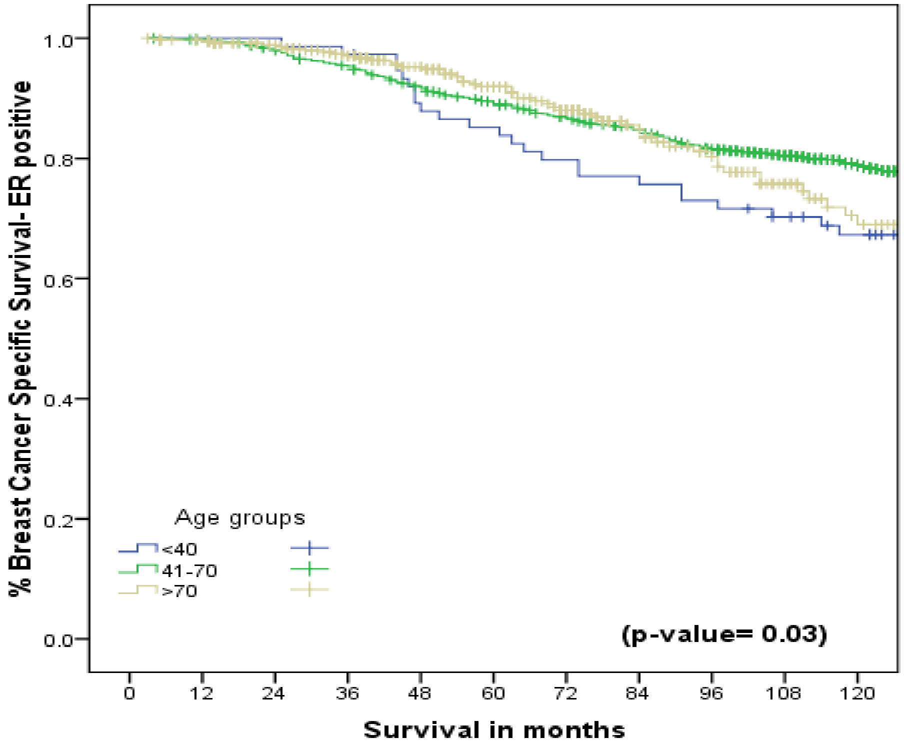

S1-b. Breast cancer specific survival Oestrogen receptor positive– comparison of age groups <40, 41-70 years and >70 years

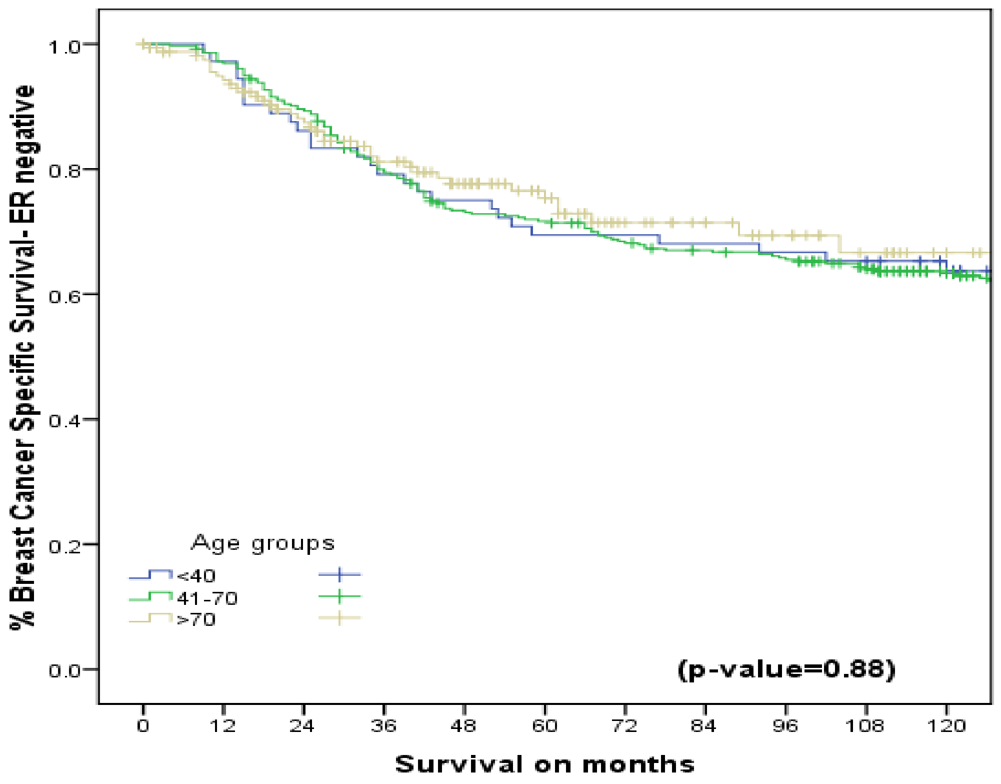

S1-c. Breast cancer specific survival in Oestrogen receptor negative – comparison of age groups <40, 41-70 years and >70 years

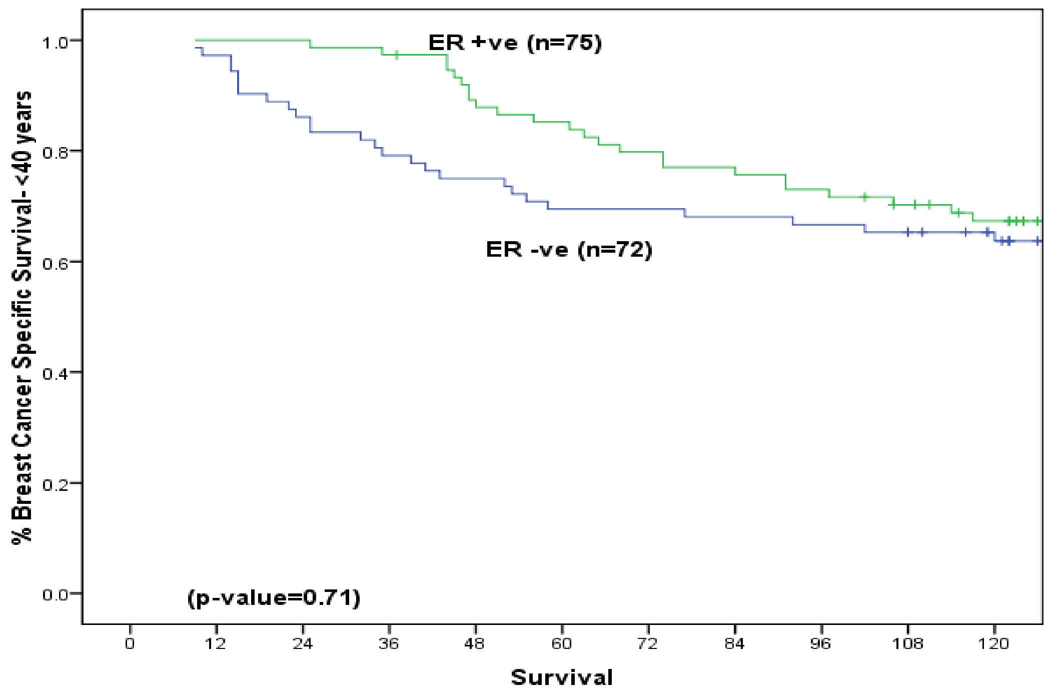

S1-d. Breast cancer specific survival <40 years – comparison of oestrogen negative and oestrogen receptor positive groups

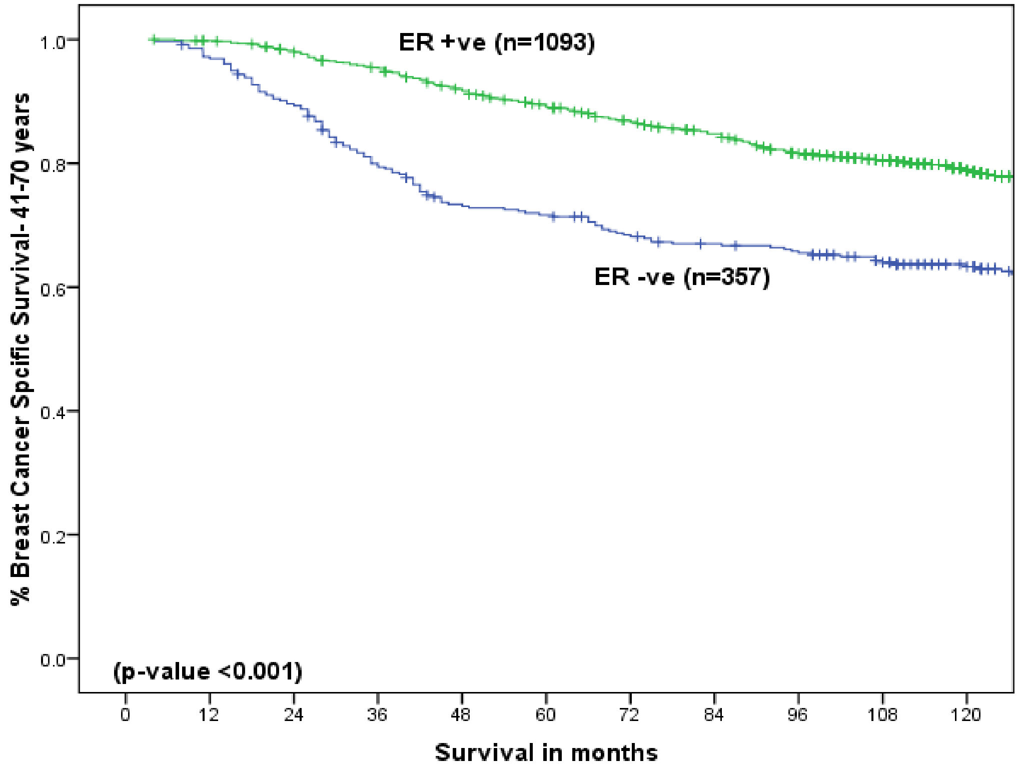

S1-e. Breast cancer specific survival 41-70 years – comparison of oestrogen negative and oestrogen receptor positive groups

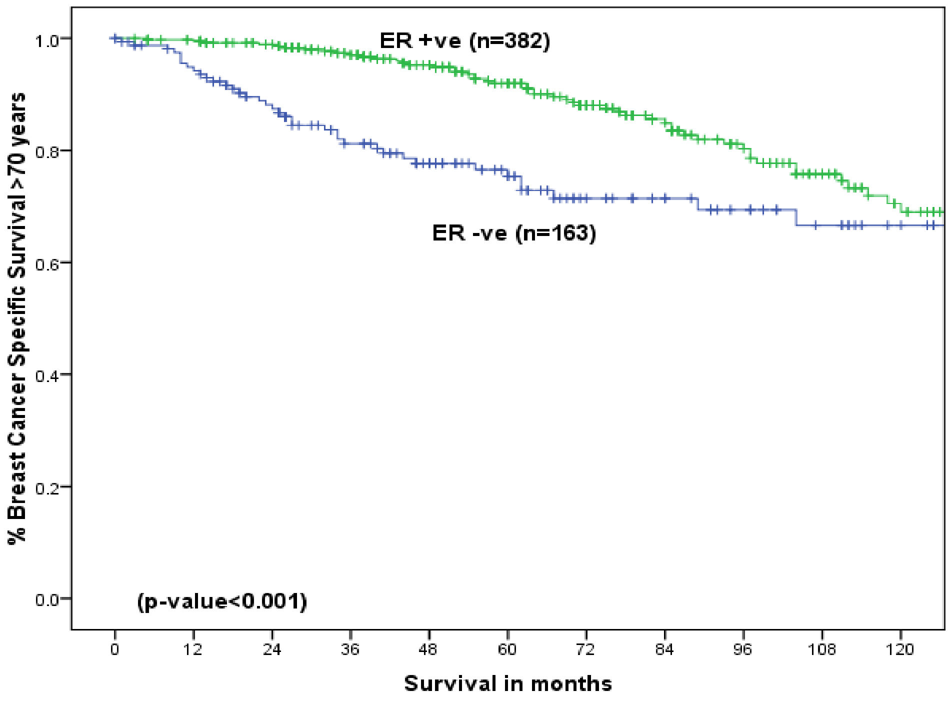

S1-f. Breast cancer specific survival >70 years – comparison of oestrogen negative and oestrogen receptor positive groups

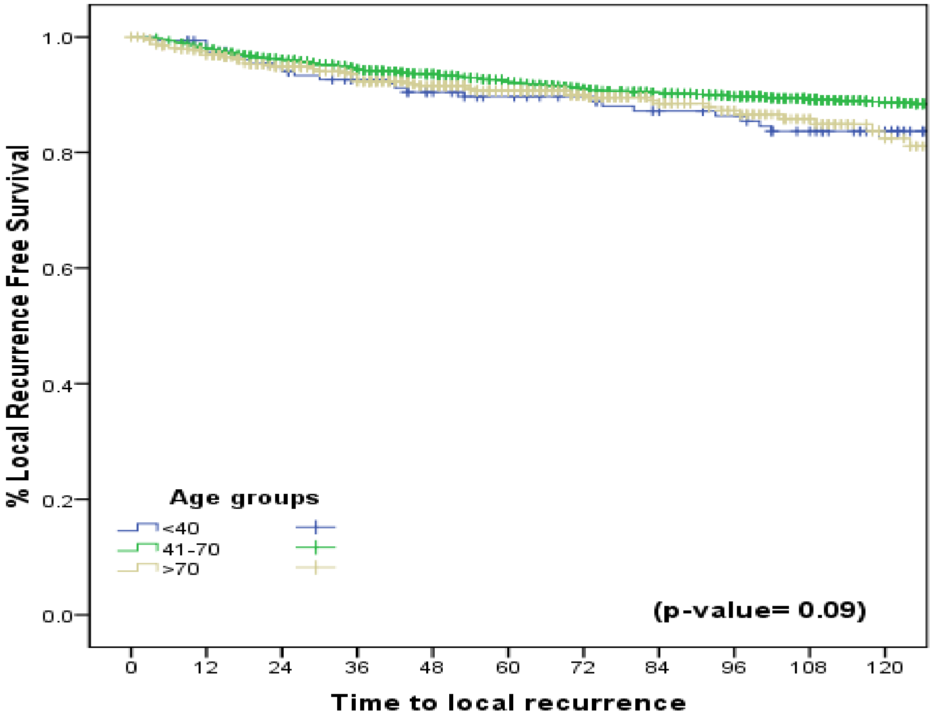

S1-g. Local recurrence free survival – comparison of age groups <40, 41-70 years and >70 years

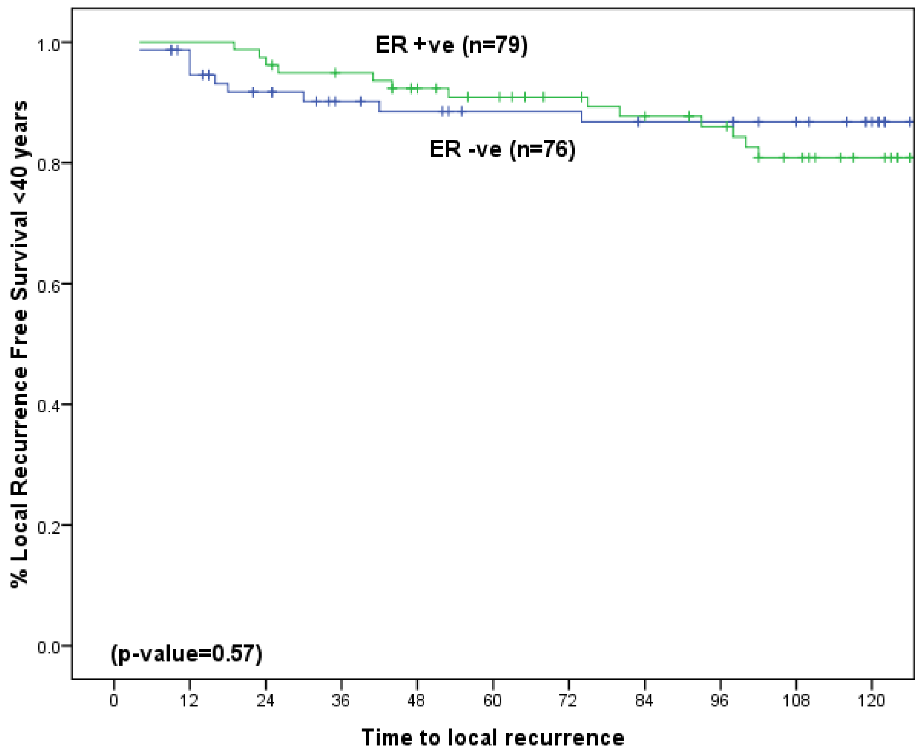

S1-h. Local recurrence free survival <40 years – comparison of oestrogen negative and oestrogen receptor positive groups

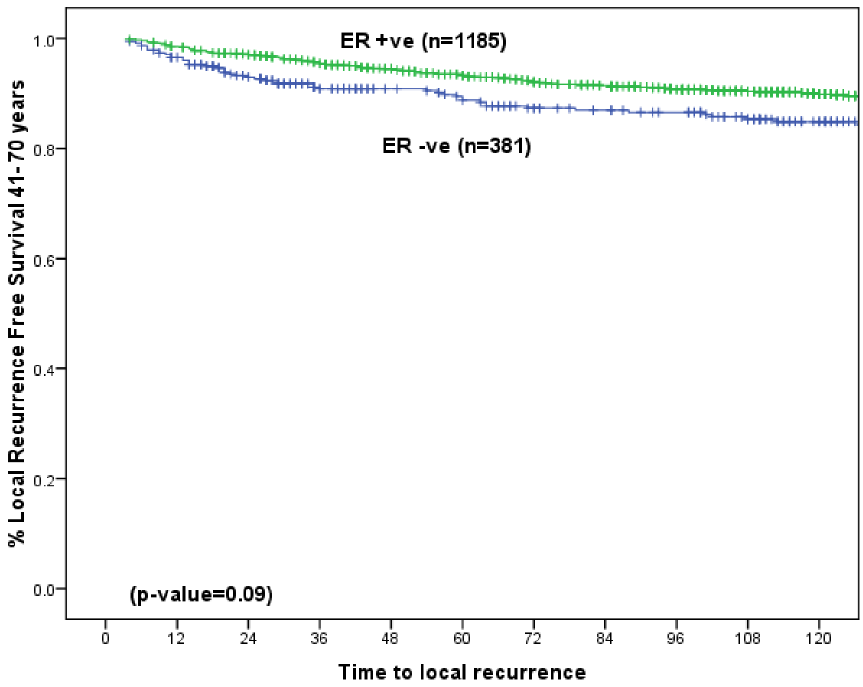

S1-i. Local recurrence free survival 41-70 years – comparison of oestrogen negative and oestrogen receptor positive groups

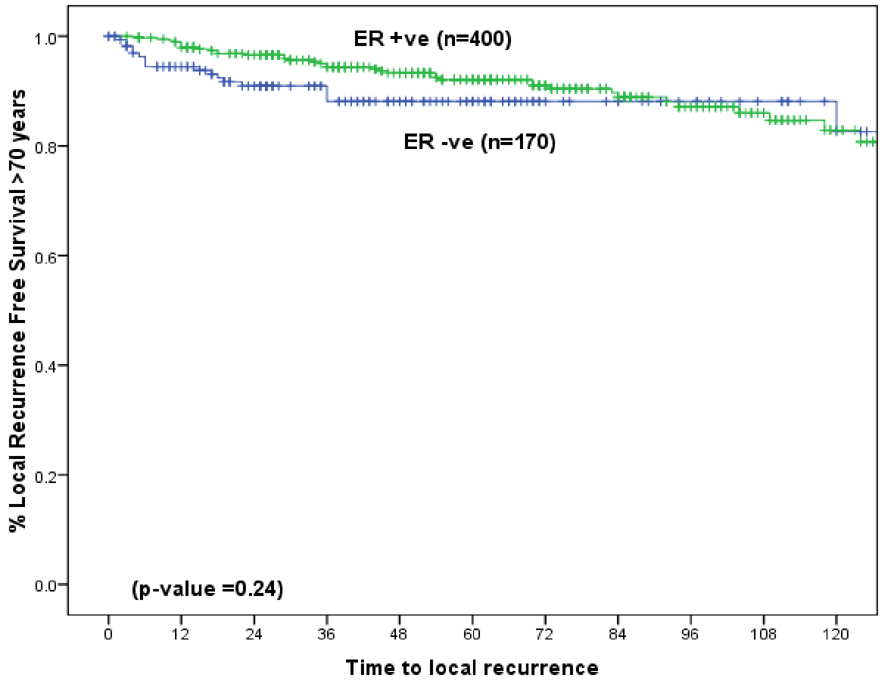

S1-j. Local recurrence free survival >70 years – comparison of oestrogen negative and oestrogen receptor positive groups

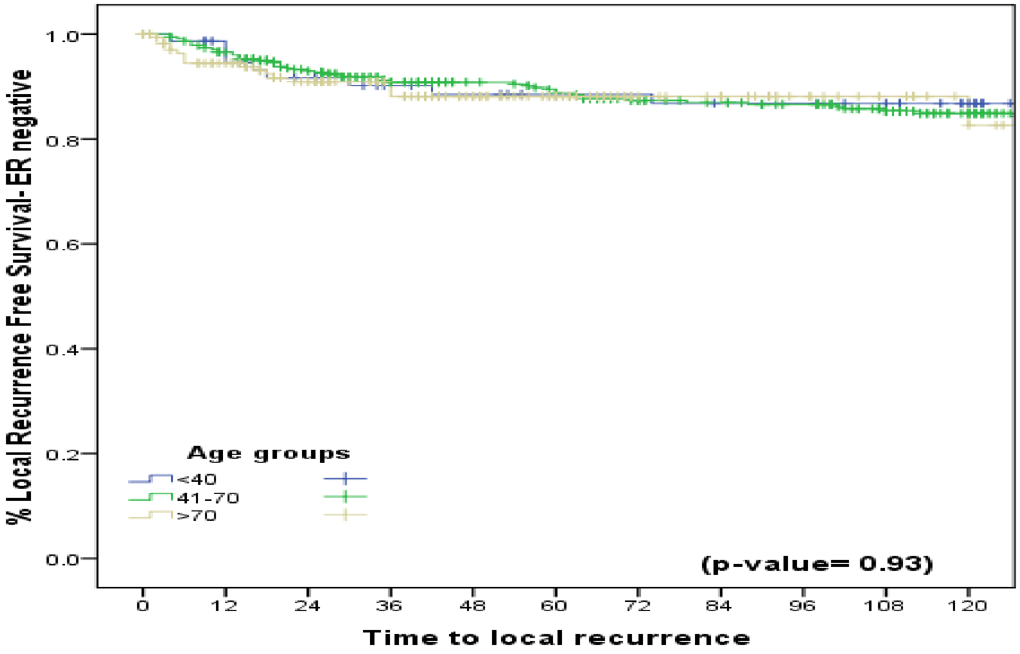

S1-k. Local recurrence free survival Oestrogen receptor positive– comparison of age groups <40, 41-70 years and >70 years

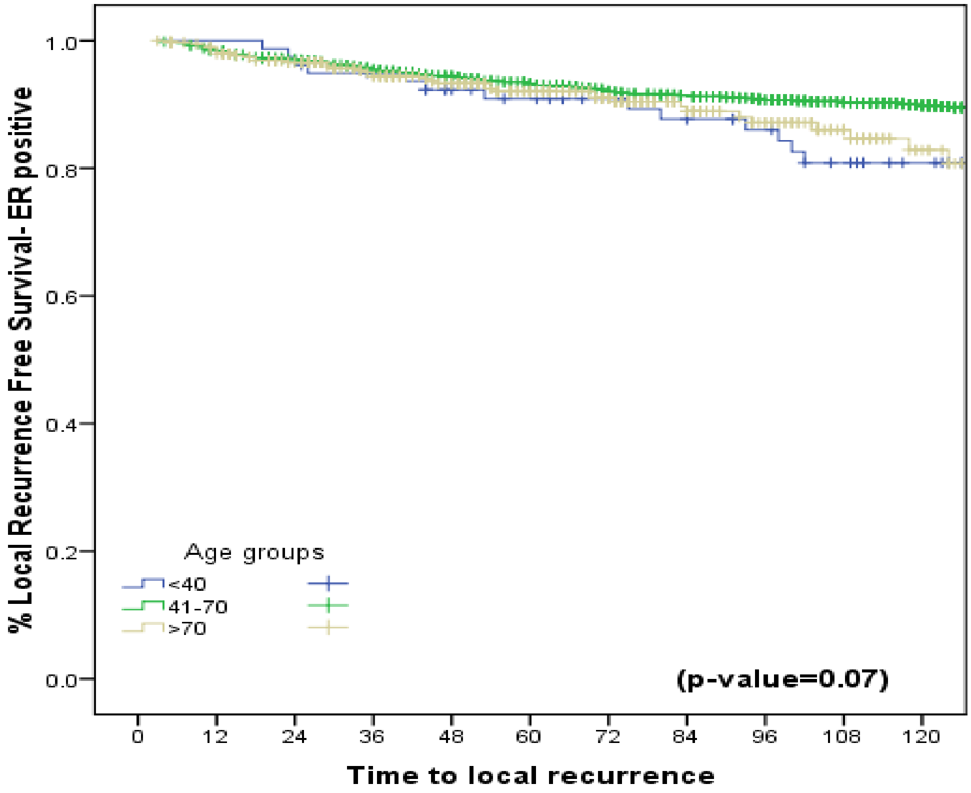

S1-l. Local recurrence free survival Oestrogen receptor negative– comparison of age groups <40, 41-70 years and >70 years

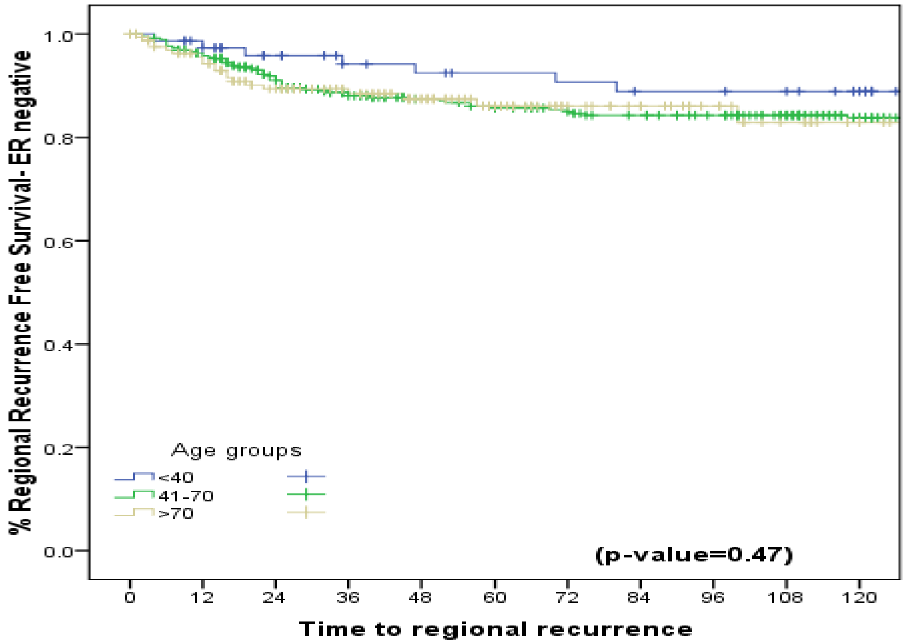

S1-m. Regional recurrence free survival Oestrogen receptor negative– comparison of age groups <40, 41-70 years and >70 years

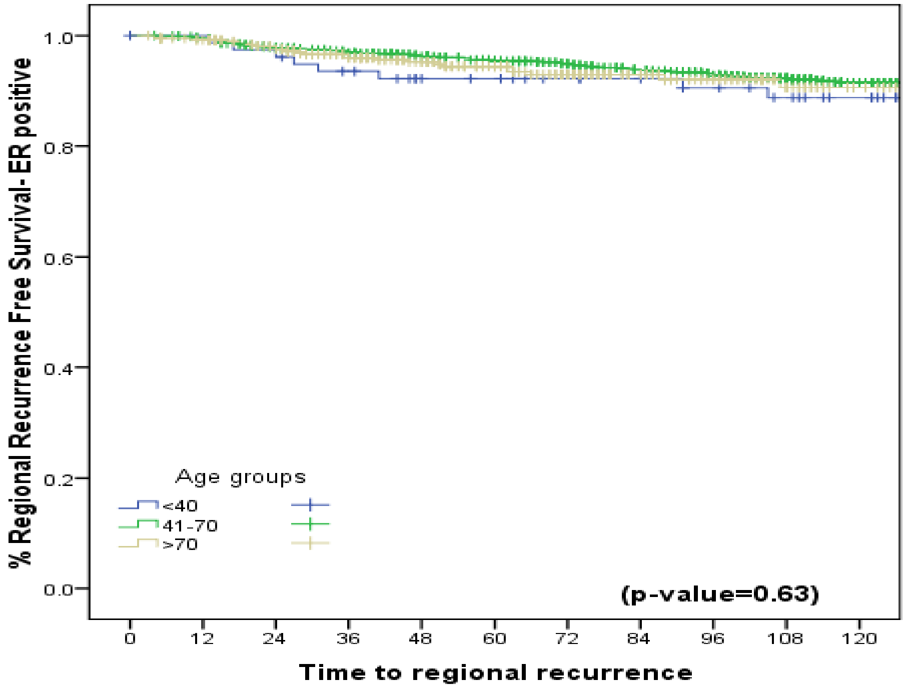

S1-n. Regional recurrence free survival Oestrogen receptor positive– comparison of age groups <40, 41-70 years and >70 years

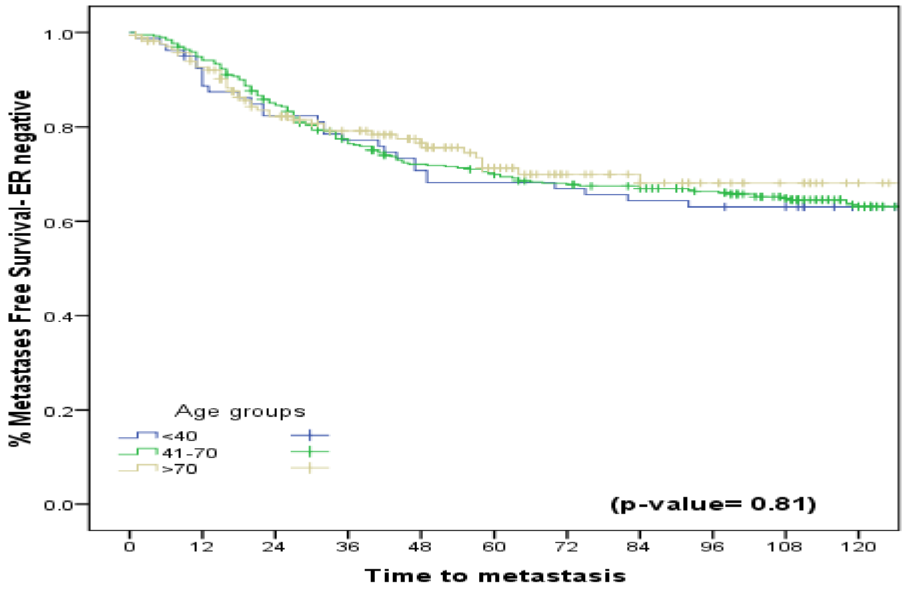

S1-o. Metastases free survival Oestrogen receptor negative– comparison of age groups <40, 41-70 years and >70 years

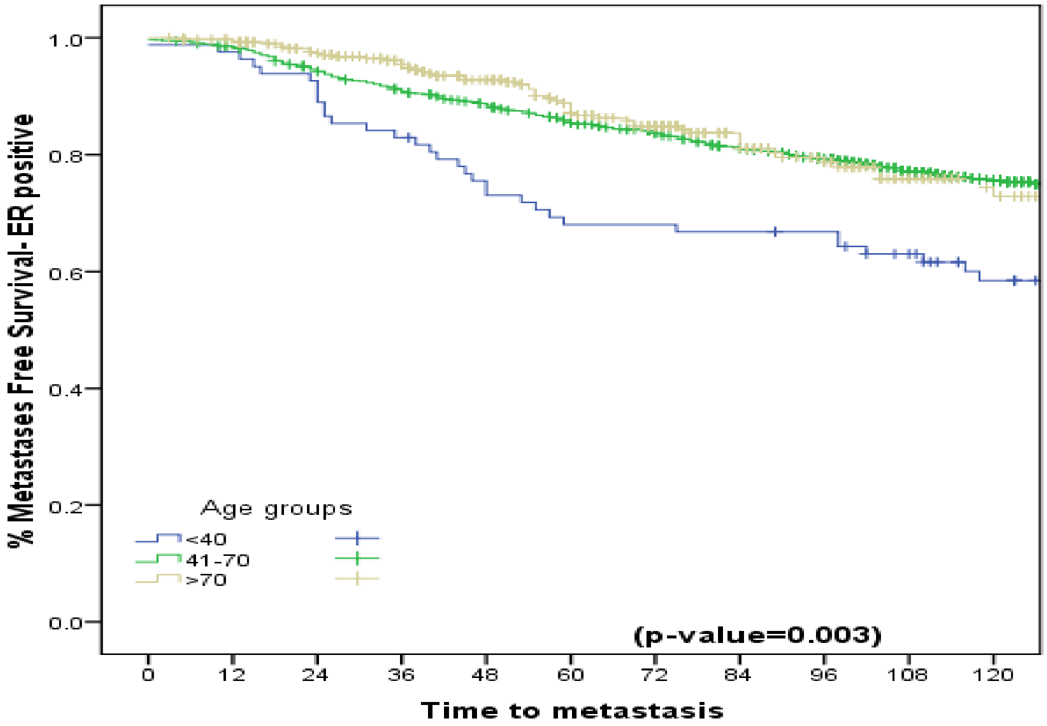

S1-p. Metastases free survival Oestrogen receptor positive– comparison of age groups <40, 41-70 years and >70 years
